# Applying a gender lens to global health and well-being: Framing a *Journal of Global Health* special collection

**DOI:** 10.7189/jogh.10.01013

**Published:** 2020-06

**Authors:** Lindsay Stark, Ilana Seff, Ann Weber, Gary L Darmstadt

**Affiliations:** 1 Brown School at Washington University in St. Louis, St. Louis, Missouri, USA; 2 Columbia University Mailman School of Public Health, New York, New York, USA; 3 School of Community Health Sciences, University of Nevada, Reno, Nevada, USA; 4 Department of Pediatrics, Stanford University School of Medicine, Stanford, California, USA

Sex and gender are widely recognised as two distinct, though related, constructs. While sex denotes an individual’s biological presentation as male, female, or intersex, gender represents the socially and culturally constructed behaviours, roles, rights, and characteristics associated with being male or female [1]. The sets of expectations and ideas about how women and men should think, act, and interact, are called gender norms, and they shape human development and health across the life-course and around the globe [2].

In addition to dictating behaviours and expectations for males and females, many prescribed gender norms engender inherent power imbalances between the two groups; in most societies, masculinity—and not femininity—is associated with higher status and value [3]. Power relations are seen by many gender theorists as central to an unequal and harmful gender system and to the expression of gender inequalities and restrictive gender norms [1]. Norms can keep the disadvantaged from challenging an unjust power distribution; they can discourage people’s self-actualisation or can be the cause of punishment for those who refuse to comply. For example, men who exhibit feminine characteristics, women who behave in masculine ways, and those who identify as a gender minority often face discrimination, marginalisation and other harms [4,5]. Further, deeply embedded gender norms are resistant to change. People’s normative beliefs are reinforced by what they witness as typical and appropriate behaviours for men and women. Even when new models and opportunities for deviating from a norm arise, people may prefer to remain within the boundaries of the gender norm, to avoid the risk of being isolated, ostracised or harmed. Importantly, the expression of gender norms is highly contextual and how people respond to norms can vary across the life course, cultures, generations, socio-economic context, and populations.

As a result of the historical legacy of gender-based injustice, “the health consequences of gender inequality fall most heavily on women, especially poor women, but restrictive gender norms undermine the health and wellbeing of women, men, and gender minorities” [6]. Gender norms can be created or reinforced at any level of the social ecology and may influence health along five gendered pathways: gendered differences in exposure, gendered health behaviors, gendered impacts on accessing care, gender-biased health systems, and gender-biased health research, institutions, and data collection. Although causal estimates of the impact of gender norms on health are scarce due to the lack of necessary data, the evidence pointing to the associations between gender norms and health outcomes is strong [2]. For example, norms “that deprive women of their autonomy and bargaining power in the family or reduce the private returns of investments in girls,” as contained in the Social Institution and Gender Index (SIGI) subcomponents, correlate with higher child mortality, higher fertility, lower secondary schooling, and more corrupt governance, even accounting for impacts of the political system and level of economic development [7]. Unequal gender norms at relational and individual levels have also been found to have consequences for health outcomes. For instance, in South Africa, men endorsing more inequitable gender norms, based on the Gender Equitable Men’s Scale and the Gender Role Conflict/Stress Scales, had higher rates of concurrent partners, intimate partner violence (IPV) perpetration, and alcohol abuse, all of which are known to increase the risk of HIV transmission [8].

This special collection in the *Journal of Global Health* builds on the evidence base linking gender norms and health, presenting findings to advance our thinking about these interconnected systems [1,2,6,9,10]. The collection takes a general focus on three areas: (1) sexual and reproductive health, (2) adolescent populations, and (3) health policy.

Three of the articles in this special collection showcase potentially simple and effective ways to improve maternal, child, and reproductive health. Omidakhsh et al. draw on national-level data sets to demonstrate how changes in national child marriage laws improve attitudes about domestic violence and reduce IPV at scale. Using similar data sources, Ríos-Salas and Heymann show how paid parental leave policies increase women’s decision-making power and improve women’s reproductive health. Finally, Dehinga et al. analyse cross-sectional data from India to show how support by a husband or family has important implications for the success of local community health workers supporting maternal and child health. Together, these three articles help advance the evidence showing how gender progressive policies and support systems can lead to improved outcomes for maternal, child, and reproductive health.

A second sub-set of articles presented in this special collection examines the ways in which attitudes and norms among relevant reference groups can influence adolescents’ behavior and the ways in which they internalise these behaviors. Shakya et al., for example, find through analysis of social network data from Honduras that a strong driver of adolescent childbirth is the frequency of adolescent childbirth both within the greater community and within an adolescent girl’s proximal social network; the important role of fathers’ presence is also highlighted. Mejía-Guevara et al. examine Demographic and Health Surveys data from nine countries in sub-Saharan Africa. They find that collective permissive attitudes of both adolescent and adult women towards premarital sex increases the use of, and demand for, contraception. In contrast, collective accepting attitudes towards wife-beating are negatively associated with the use of, and demand for, contraception. Stark et al. examine the impact of perpetrating IPV on the perpetrator’s mental health among adolescents and young adults in Nigeria. While males are more likely than females to perpetrate IPV, both male and female perpetrators report negative, but different, mental health outcomes, suggesting that socially determined gender norms may shape the ways in which distress from IPV perpetration is understood and expressed. Together, these articles not only identify ways in which gender norms may advance or impede progress in achieving the global health agenda, but also suggest specific groups that may be strategically targeted in order to promote and foster change among adolescents.

In two final articles in this collection, the authors present innovative ways of looking at the relationships between gender norms and health and deriving insights for policy formulation. First, Cislaghi et al. offer a new approach for analysing causes of disability-adjusted life years to help policy makers distinguish drivers that may be rooted in biology as opposed to those that may be rooted in harmful gender systems. Such analyses call on expanding systems of social and gender justice alongside more traditional medical and public health approaches. Second, Shawar and Shiffman critically examine structural barriers affecting global health organisations in addressing gender inequality. Their thematic analysis identifies three major challenges currently impeding success: 1) a lack of cohesion among health champions, 2) differences concerning the nature of the problem and solutions, including whether reducing gender inequality or addressing harmful gender norms is the primary goal, and 3) disagreements among proponents on how to convey the problem. The authors conclude that the success of the global health agenda will depend in part on the ability of proponents to address these disagreements and develop strategies for negotiating complex organisational cultures and political environments.

Taken together, the articles that make up the special collection advance our thinking around gender norms and health in important ways. First, the collection underscores how gender norms operate across the lifecycle, from infancy and early childhood into adolescence and throughout adulthood. It is never too early nor too late in the lifecycle to start thinking about how gender norms are affecting health. Second, the collection shows how norms operate at communal and relational levels all the way up to national policy frameworks. This finding supports our ability to make positive changes at multiple levels within a society to benefit population health. Finally, the collection shows how medicine and public health cannot focus on biological causes and mechanisms without also addressing social factors to achieve positive health outcomes.

Women’s equality and empowerment is one of the 17 Sustainable Development Goals (SDGs), and addressing harmful gender norms for both males and females is integral to all dimensions of inclusive and sustainable development. In order to have truly sustainable development and health, the global community needs to create programmes and policies that seek to transform restrictive gender norms and promote equality. Despite the growing body of literature that illustrates the degree to which gender norms significantly impact all pathways to well-being, there is a need for greater coordination and collaboration of gendered intervention work across the development community. To accurately understand the multidimensional ways in which gendered systems influence the pathways to achieving the SDGs, there needs to be substantially greater investment in gender-focused research, policy development, and action.

## References

[1] Heise L Greene ME Opper N Stavropoulou M Harper C Nascimento M Gender inequality and restrictive gender norms: Framing the challenges to health. Lancet. 2019;393:2440-54. 10.1016/S0140-6736(19)30652-X 31155275

[2] Weber AM Cislaghi B Meausoone V Abdalla S Mejía-Guevara I Loftus P Gender norms and health: Insights from global survey data. Lancet. 2019;393:2455-68. 10.1016/S0140-6736(19)30765-2 31155273

[3] Ridgeway CL Correll SJ Unpacking the gender system: A theoretical perspective on gender beliefs and social relations. Gend Soc. 2004;18:510-31. 10.1177/0891243204265269

[4] Shakya HB Domingue B Nagata JM Cislaghi B Weber AM Darmstadt GL Adolescent gender norms and adult health outcomes in the USA: A prospective cohort study. Lancet Child Adolesc Health. 2019;3:529-38. 10.1016/S2352-4642(19)30160-9 31155319 PMC6686658

[5] Domingue BW Cislaghi B Nagata JM Shakya HB Weber AM Boardman JD Implications of gendered behaviour and contexts for social mobility in the USA: A nationally representative observational study. Lancet Planet Health. 2019;3:e420-8. 10.1016/S2542-5196(19)30191-3 31625514 PMC6876275

[6] Gupta GR Oomman N Grown C Conn K Hawkes S Shawar YR Gender equality and gender norms: Framing the opportunities for health. Lancet. 2019;393:2550-62. 10.1016/S0140-6736(19)30651-8 31155276

[7] Branisa B Klasen S Ziegler M Gender inequality in social institutions and gendered development outcomes. World Dev. 2013;45:252-68. 10.1016/j.worlddev.2012.12.003

[8] Gottert A Barrington C McNaughton-Reyes HL Maman S MacPhail C Lippman SA Gender norms, gender role conflict/stress and HIV risk behaviors among men in Mpumalanga, South Africa. AIDS Behav. 2018;22:1858-69. 10.1007/s10461-017-1706-9 28161801 PMC6440537

[9] Heymann J Levy JK Bose B Ríos-Salas V Mekonen Y Swaminathan H Improving health with programmatic, legal, and policy approaches to reduce gender inequality and change restrictive gender norms. Lancet. 2019;393:2522-34. 10.1016/S0140-6736(19)30656-7 31155271

[10] Hay K McDougal L Percival V Henry S Klugman J Wurie H Disrupting gender norms in health systems: Making the case for change. Lancet. 2019;393:2535-49. 10.1016/S0140-6736(19)30648-8 31155270 PMC7233290

